# Anisotropic in-plane spin splitting in an asymmetric (001) GaAs/AlGaAs quantum well

**DOI:** 10.1186/1556-276X-6-520

**Published:** 2011-09-02

**Authors:** Huiqi Ye, Changcheng Hu, Gang Wang, Hongming Zhao, Haitao Tian, Xiuwen Zhang, Wenxin Wang, Baoli Liu

**Affiliations:** 1 Beijing National Laboratory for Condensed Matter Physics, Institute of Physics, Chinese Academy of Sciences, P.O. Box 603, Beijing 100190, China; 2 College of Physics, Jilin University, Changchun, 130021, China; 3 State Key for Superlattices and Microstructures, Institute of Semiconductors, Chinese Academy of Sciences, P.O. Box 912, Beijing 100083, China

**Keywords:** quantum beats, spin-orbit coupling, magnetic properties of nanostructures, optical creation of spin polarized

## Abstract

The in-plane spin splitting of conduction-band electron has been investigated in an asymmetric (001) GaAs/Al*_x_*Ga_1-*x*_As quantum well by time-resolved Kerr rotation technique under a transverse magnetic field. The distinctive anisotropy of the spin splitting was observed while the temperature is below approximately 200 K. This anisotropy emerges from the combined effect of Dresselhaus spin-orbit coupling plus asymmetric potential gradients. We also exploit the temperature dependence of spin-splitting energy. Both the anisotropy of spin splitting and the in-plane effective *g*-factor decrease with increasing temperature.

**PACS: **78.47.jm, 71.70.Ej, 75.75.+a, 72.25.Fe,

## Introduction

The properties of spins in semiconductor materials have attracted much more attentions since the invention of spintronics and spin-based quantum information [1-3]. In those fields, the spin-orbit coupling (SOC) plays a key role on the properties of spin states in bulk and low-dimensional semiconductor materials. It not only results in the zero-magnetic field spin splitting, which is the main source of the spin relaxation through D'yakonov-Perel (DP) mechanism and novel phenomenon such as the generation of the spin current [4], but also significantly affects the spin splitting with an external magnetic field *B *> 0.

In general, the spin splitting of electron or hole at *B *> 0 in semiconductor is described by a finite Zeeman splitting energy and characterized by the effective *g*-factor, which is necessary for the spin manipulation and spin-based qubit with an external electrical/magnetic field in semiconductor. So far, the effective *g*-factor has been intensively investigated in many literatures during past few decades [5-13]. For conduction-band electron, it is found that the effective *g*-factor is strongly dependent on the point group symmetry in semiconductor materials [7]. It is isotropic and independent on the orientation of applied magnetic field in GaAs bulk due to *T*_d _point symmetry group. On the contrast, the effective *g*-factor becomes anisotropic and significantly depends on the direction of magnetic field in quantum structures such as GaAs/AlGaAs heterostructures and quantum well (QW) due to the reducing symmetry [7]. For example, where the point symmetry group is reduced to *D*_2d_, in a rectangular/symmetric QW grown on the (001)-orientated substrate, the effective *g*-factor can have different values for *B *applied in the direction normal to the plane of QW and for *B *in the plane of the QW due to the additional potential confinement: *g_xx _*= *g_yy _*≠ *g_zz _*(*x*//[100]) [5-7,10]. Furthermore, where the symmetry is reduced to *C*_2v _in an asymmetric QW with the inversion-asymmetric confining potentials, the effective *g*-factor is dependent on the direction of an applied in-plane magnetic field, which results in the anisotropic Zeeman splitting [14]. Up to now, the spin splitting (Zeeman splitting) at *B *> 0 is considered to be only characterized by the effective *g*-factor. In fact, two proposals [7,14] have been predicted that the Dresselhaus SOC significantly affects the spin splitting of electron at *B *> 0 plus structure inversion asymmetry. A new term, defined as $b_{41,2}^{6c6c}$ in Ref. [14], can result in the measurable anisotropy of the in-plane spin splitting, although it is not a Zeeman term. We call it as Zeeman-like term thereinafter. The anisotropic spin splitting was measured experimentally at *B *> 0 with an applied external electric field to reduce the symmetry of quantum film but interpreted in terms of anisotropic effective *g*-factor by Oestreich et al. [9] In this Letter, we use the time-resolved Kerr rotation (TRKR) [15,16] technique to study the in-plane spin splitting via the accurate measurements of the Larmor procession frequency in a specially designed (001) GaAs/AlGaAs undoped QW with asymmetric confined barriers under an in-plane magnetic field. We show that the spin splitting is found to be obviously anisotropic for *B *parallel to [110] and [1-10].

## Experimental procedure

The sample on investigation here is grown on (001) oriented semi-insulating GaAs substrate by molecular beam epitaxy. It contains a 50-nm-wide Al_0.28_Ga_0.72_As barrier layer, an 8-nm-wide GaAs quantum well, the other sloping barrier grown with content of Al changing from 4.28% to 28% on the length of approximately 9 nm, and the barrier layer of a width 50 nm. The upper part of the structure is covered with a 5-nm GaAs layer to avoid the oxidation of barrier. TRKR experiment was carried out in an Oxford magneto-optical cryostat supplied with a 7-T split-coil super-conducting magnet. The sample was excited near normal incidence with a degenerate pump and delayed probe pulses from a Coherent mode-locked Ti-sapphire laser (approximately 120 fs, 76 MHz). The center of the photon energy was tuned for the maximum Kerr rotation signal for each temperature setting. The laser beams were focused to a spot size of approximately 100 μm, and the pump and probe beams have an average power of 5.0 and 0.5 mW, respectively. The helicity of linearly polarized pump beam was modulated at 50 kHz by a photoelastic modulator for lock-in detection. The temporal evolution of the electron spins, which were generated by the circularly polarized pump pulse, was recorded by measuring Kerr rotation angle *θ*_K_(Δ*t*) of the linearly polarized probe pulse while sweeping Δ*t*.

## Results and discussion

**Figure 1:**
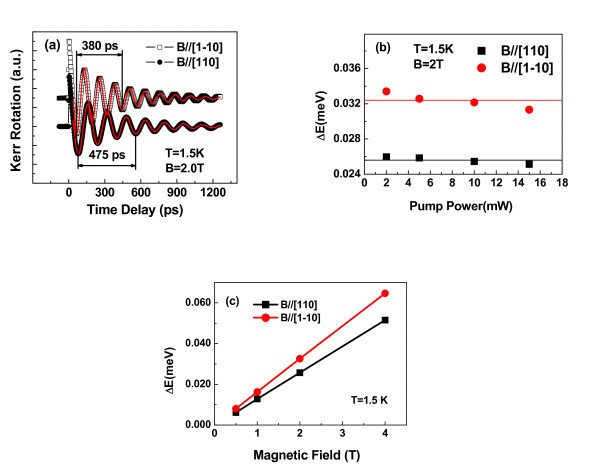
**Time-resolved Kerr rotation measurements and pump power dependence of spin splitting**. (**a**) Time-resolved Kerr rotation measurements in an asymmetric (001) QW sample for a magnetic field *B *= 2 T along [110] and [1-10], respectively, at *T *= 1.5 K. The red lines are the fitting curves. (**b**) Pump power dependence of spin splitting for *T *= 1.5 K and *B *= 2 T. The solid line presents the average value of spin splitting of all pump powers. (**c**) The spin splitting as a function of magnetic field at 1.5 K. (Color online).

Figure 1a shows the time evolution of the Kerr rotation *θ*_K_(Δ*t*) measured at 1.5 K with an in-plane magnetic field of *B *= 2.0 T (Voigt geometry [3]). The experimental data are plotted by open rectangular and solid circular symbols, which represent that the magnetic fields are applied along axes [110] and [1-10], respectively. The data show strong oscillations corresponding to the spin precession about the external magnetic field. Here, the affect of hole spin is ignored due to fast spin relaxation [17]. It is obvious that Larmor precession periods of two curves are different. The duration of three precession periods, as labeled in Figure 1a, corresponds to 3*T*_L _= 475 and 380 ps for *B*//[110] and [1-10], respectively. The experimental spin procession dynamics are well fitted with a mono-exponential decay time and a single frequency as presented by red lines in Figure 1a by the following equation:

(1) S⊥(t)=S0 exp(-t∕τs)cos(ωt),

where *S*_0 _is the initial spin density, τ_s _is spin lifetime, and *ω *the Larmor frequency. By this way, we obtain the exact value of the Larmor frequency ω and then the splitting energy Δ*E_B//[110] _*= 0.0263 meV and Δ*E_B//[1-10] _*= 0.0326 meV through the equation Δ*E *= *ћω *with in-plane magnetic field parallel to [110] and [1-10], respectively. Here, we use $\left|\Delta E_{\left[1\bar{1}0\right]}-\Delta E_{\left[110\right]}\right|∕\left|\Delta E_{\left[1\bar{1}0\right]}\right|$ to denote the anisotropy of the in-plane spin splitting. We found that this anisotropy is more than 19% in this single asymmetric (001) GaAs/AlGaAs QW. We also checked the photogenerated spin concentration dependence of the spin splitting, which can be reached by changing the pump power. Figure 1b shows the pump power dependence of spin splitting with the magnetic fields along [110] and [1-10] at 1.5 K. The splitting energy slowly decreases with increasing pump power up to approximately 20 mW. The change of spitting energy is less than 7% for both curves and can be ignored. Therefore, the observed anisotropy is not relevant to the carrier concentration.

Now we extract the contribution of Zeeman-like term $b_{41,2}^{6c6c}$ on the anisotropic in-plane spin splitting at *B *> 0. As calculated by Winkler, the spin-splitting energy writes as [14]:

(2) $$\Delta E=GB_{∕∕}=\left(g^{*}\mu_{B}-2\zeta b_{41,2}^{6c6c}\right)B_{∕∕}$$

(3) $$b_{41,2}^{6c6c}=\frac{e}{\hbar }\gamma \left(\left\langle k_{z}^{2}\right\rangle \left\langle z\right\rangle -\left\langle \left\{k_{z}^{2},z\right\}\right\rangle \right)$$

where g^* ^is the effective *g*-factor, *B*_// _is the in-plane external magnetic field, ζ = +1 for ***B***// [1-10] and ζ = -1 for *B*//[110], and *γ *is the cubic Dresselhaus constant. The Zeeman-like term $b_{41,2}^{6c6c}$, which is derived from first-order perturbation theory applied to the Dresselhaus term, emerges from the combined effect of BIA and SIA [14]. It is clear that the term $b_{41,2}^{6c6c}$ results in the anisotropic spin splitting at *B *> 0. As expected in Equation 2, the measured spin splitting is linearly dependent on the magnetic field with a prefactor $G=g^{*}\mu_{B}-2\zeta b_{41,2}^{6c6c}$ for both directions of applied magnetic fields as shown in Figure 1c. The slope of the *B *linear dependence will allow us to obtain the value of *G *accurately, which are *G*_[110] _= 0.0130 meV/T and $G_{\left[1\bar{1}0\right]}$ = 0.0162 meV/T for *B *along [110] and [1-10]. The difference of two values results from the opposite sign of prefactor *ζ*. According to Equation 3, $b_{41,2}^{6c6c}$ is found to be equal to approximately 0.8 μeV/T. As discussed above, a proper anisotropic Zeeman term, described in Equation (7.4) in Ref. [14], also produces the anisotropic spin splitting at *B *> 0 in an asymmetric (001) GaAs/AlGaAs QW. However, the prefactor $z_{41}^{6c6c}\varepsilon_{z}$ is about 0.039 μeV/T for realistic parameters with the assumed internal electric field of approximately 50 kV/cm induced by the asymmetric potential gradients. It is about 20 times smaller comparing to the value of term $b_{41,2}^{6c6c}$. We conclude that the Zeeman-like term $b_{41,2}^{6c6c}$ is the main source of the anisotropy of spin splitting at *B *> 0 in an asymmetric QW. Additionally, the Rashba term also gives rise to a nontrivial splitting in the presence of a magnetic field, but the splitting is isotropic [14]. In fact, the Rashba term is considered to be very small in this work because we did not observe significantly the anisotropy of in-plane spin relaxation [16] as shown in Figure 1a. It is consistent with the results of Ref. [18]. As shown in Equation 3, the Zeeman-like term $b_{41,2}^{6c6c}$ is proportional to the cubic Dresselhaus constant *γ*. Numerical calculations yields *γ *= 29.96 eV/Å^3 ^at approximately 1.5 K. Here, we use the value of approximately 0.8 μeV/T of $b_{41,2}^{6c6c}$ and an electron wave function calculated by the k p method [19] in this asymmetric QW. It is in agreement with the value of 27.58 eV/Å^3 ^(see Table 6.3 in Ref. [14]). The remaining deviations of *γ *probably result from differences between the actual and the nominal sample structures which lead to uncertainties in the calculation of the wave function asymmetry.

**Figure 2:**
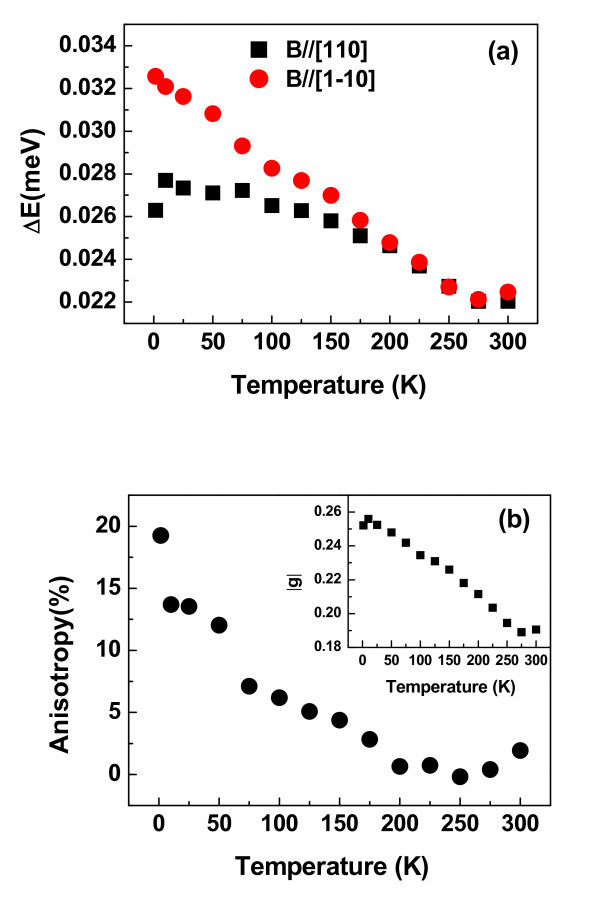
**The temperature dependence of spin splitting and the anisotropy**. The temperature dependence of (**a**) the spin splitting for *B*//[110] and [1-10], respectively; (**b**) the anisotropy (Δ*E_B//[1-10] _*- Δ*E_B//[110]_*)/ΔE*_B//[1-10]_*. The inset of (b) shows the in-plane effective *g*-factor as a function of temperature. (Color online).

Finally, we systematically investigate the anisotropy of in-plane spin splitting for the temperatures between 1.5 and 300 K keeping the fixed excitation power of approximately 5 mW and the fixed external magnetic field of approximately 2 T. Figure 2a shows the values of spin splitting as a function of temperature for *B *along [110] and [1-10], respectively. Both values decrease while the temperature is elevated. It is noted that the difference of spin splitting is maximum at low temperature of approximately 1.5 K and almost disappears when the temperature is up to 200 K. In order to clearly show the anisotropy of spin splitting, we have extracted precisely the values of $\left|\Delta E_{\left[1\bar{1}0\right]}-\Delta E_{\left[110\right]}\right|∕\left|\Delta E_{\left[1\bar{1}0\right]}\right|$ for the full temperature range and depicted in Figure 2b. As discussed above, the term $b_{41,2}^{6c6c}$ is dominant in the anisotropic spin splitting at *B *> 0. Let us recall the expression of prefactor $b_{41,2}^{6c6c}$, the electron is implied to be phase coherent before colliding with the walls. This assumption is true at low temperature. However, the phase coherent length of electron is not a constant while the temperature varies from 1.5 to 300 K [20,21]. We believe this is main source of decreasing of the spin-splitting anisotropy with increasing temperature. The in-plane effective electron *g*-factor can also be extracted from Equation 2. It is about g^* ^= 0.25 at 1.5 K and very closed to that (g^* ^= 0.26) in 10-nm-width well with the same Al fraction [11]. The inset of Figure 2b shows temperature dependence of in-plane effective electron *g*-factor. It decreases with increasing temperature. This trend is consistent with the former reports [8,12,13].

## Conclusions

We observed the anisotropic in-plane spin splitting of the conduction-band electron in an asymmetric (001) GaAs/AlGaAs quantum well using TRKR technique with applied magnetic fields. It is confirmed that Dresselhaus spin-orbit coupling can significantly affect the in-plane spin splitting at *B *> 0 combining the asymmetric confinement potential via the numerical comparison with the proper anisotropic Zeeman splitting.

## Abbreviations

BIA: bulk inversion asymmetry; DP: D'yakonov-Perel; QW: quantum well; SIA: structure inversion asymmetry; SOC: spin-orbit coupling; TRKR: time-resolved Kerr rotation.

## Competing interests

The authors declare that they have no competing interests.

## Authors' contributions

BL conceived and designed the experiments. HQ and CC carried out the experiments with contribution from GW and HM. HT and WX provided the sample. ZX contributed to the calculation. BL supervised the work. HQ and BL wrote the manuscript. All authors read and approved the final manuscript.

### Open access

This article is distributed under the terms of the Creative Commons Attribution Noncommercial License which permits any noncommercial use, distribution, and reproduction in any medium, provided the original author(s) and source are credited.

## References

[B1] Fabian J Matos-Abiague A Ertler C Stano P Žutić I Semiconductor spintronics Acta Physica Slovaca 2007 57 565 10.2478/v10155-010-0086-8

[B2] Awschalom DD Loss D Samarth N Semiconductor Spintronics and Quantum Computation 2002 Heidelberg: Springer-Verlag

[B3] Žutić I Fabian J Das Sarma S Spintronics: fundamentals and applications Rev Mod Phys 2004 76 323 10.1103/RevModPhys.76.323

[B4] Sinova J Culcer D Niu Q Sinitsyn NA Jungwirth T MacDonald AH Universal intrinsic spin Hall effect Phys Rev Lett 2004 92 126603 15089695 10.1103/PhysRevLett.92.126603

[B5] Ivchenko EL Kiselev AA Electron *g*-factor of quantum-well and superlattices Sov Phys Semicond 1992 26 827

[B6] Kalevich VK Korenev VL Anisotropy of the electron *g*-factor in GaAs/AlGaAs quantum-wells JETP Lett 1992 56 253

[B7] Kalevich VK Korenev VL Electron *g*-factor anisotropy in asymmetric GaAs/AlGaAs quantum well JETP Lette 1993 57 557

[B8] Oestreich M Rühle WW Temperature dependence of the electron Landé *g *factor in GaAs Phys Rev Lett 1995 74 2315 10.1103/PhysRevLett.74.2315 10057897

[B9] Oestreich M Hallstein S Rühle WW Quantum beats in semiconductors IEEE J Sel Top Quantum Electron 1996 2 747 10.1109/2944.571776

[B10] Le Jeune P Robart D Marie X Amand T Brousseau M Barrau J Kalevich V Rodichev D Anisotropy of the electron Landé *g *factor in quantum wells Semicond Sci Technol 1997 12 380 10.1088/0268-1242/12/4/006

[B11] Yugova IA Greilich A Yakovlev DR Kiselev AA Bayer M Petrov VV Dolgikh YuK Reuter D Wieck AD Universal behavior of the electron g factor in GaAs/Al*_x_*Ga_1-*x*_As quantum wells Phys Rev B 2007 75 245302

[B12] Zawadzki W Pfeffer P Bratschitsch R Chen Z Cundiff ST Murdin BN Pidgeon CR Temperature dependence of the electron spin g factor in GaAs Phys Rev B 2008 78 245203

[B13] Hübner J Döhrmann S Hägele D Oestreich M Temperature-dependent electron Landé *g *factor and the interband matrix element of GaAs Phys Rev B 2009 79 193307

[B14] Winkler R Spin-Orbit Coupling Effects in 2D Electron and Hole Systems 2003 Chapter 7 Berlin: Springer

[B15] Koopmans B Haverkort JEM de Jonge WJM Karczewski G Time-resolved magnetization modulation spectroscopy: a new probe of ultrafast spin dynamics J Appl Phys 1999 85 6763 10.1063/1.370191

[B16] Liu BL Zhao HM Wang J Liu LS Wang WX Chen DM Electron density dependence of in-plane spin relaxation anisotropy in GaAs/AlGaAs two-dimensional electron gas Applied Phys Lett 2007 90 112111 10.1063/1.2713353

[B17] Amand T Marie X Le Jeune P Brousseau M Robart D Barrau J Planel R Spin quantum beats of 2D excitons Phys Rev Lett 1997 78 1355 10.1103/PhysRevLett.78.1355

[B18] Eldridge PS Leyland WJH Lagoudakis PG Harley RT Phillips RT Winkler R Henini M Taylor D Rashba conduction band spin-splitting for asymmetric quantum well potentials Phys Rev B 2010 82 045317

[B19] Xia JB Effective-mass theory for superlattices grown on (11N)-oriented substrates Phys Rev B 1991 43 9856 10.1103/PhysRevB.43.9856 9996688

[B20] Hiramoto T Hirakawa K Iye Y Ikoma T Phase coherence length of electron waves in narrow AlGaAs quantum wires fabricated by focused ion beam implantation Appl Phys Lett 1989 54 2103 10.1063/1.101177

[B21] Bäuerle C Mallet F Schopfer F Mailly D Eska G Saminadayar L Experimental test of numerical renormalization-group theory for inelastic scattering from magnetic impurities Phys Rev Lett 2005 95 266805 16486386 10.1103/PhysRevLett.95.266805

